# Occurrence of Immune Thrombocytopenic Purpura Following Enucleation of a Neuroendocrine Tumor of the Head of the Pancreas

**DOI:** 10.7759/cureus.47109

**Published:** 2023-10-16

**Authors:** Mohamed Jazeer, Chaminda Garusinghe

**Affiliations:** 1 Internal Medicine, Teaching Hospital, Batticaloa, Batticaloa, LKA; 2 Endocrinology, Colombo South Teaching Hospital, Colombo, LKA

**Keywords:** megakaryopoiesis, disseminated intravascular coagulation (dic), chromogranin-a, pancreatic neuroendocrine tumors, immune mediated thrombocytopenic purpura (itp)

## Abstract

Neuroendocrine tumors are growths occurring in various organs including the pancreas which contain endocrine tissue. Pancreatic neuroendocrine tumors are rare in occurrence with an incidence of <1 per 100,000 individuals. But the incidence is rising and the said tumors are becoming more common in the world. Herein we report a case of a 49-year-old female with a background history of poorly controlled type 2 diabetes mellitus for 7 years, dyslipidemia, and bronchial asthma for 19 years on regular inhaler therapy presenting with a history of back pain for 3 months duration. With serial investigations, she was found to have a neuroendocrine tumor involving the head of the pancreas with no local infiltration and distant metastases. Whipple’s procedure was performed after which she developed thrombocytopenia from post-operative day 1 itself. Following this, she was diagnosed as having immune thrombocytopenic purpura and being managed with oral corticosteroids, after which she made a successful recovery from the acute condition but with multiple relapses from time to time.

## Introduction

Neuroendocrine tumors are usually sporadic in nature but on the contrary, they can occur as a consequence of a germ-line mutation and can cause multiple hereditary endocrinopathies including multiple endocrine neoplasia type 1 or 4 (MEN 1 & 4), neurofibromatosis type 1, tuberous sclerosis, von Hippel Lindau disease, etc. Clinical manifestations of pancreatic neuroendocrine tumors can vary depending on the type of hormonal secretion to which the tissues are attributed. In the case of insulinoma excess insulin secretion is noted while if it is gastrinoma, glucagonoma, or vasoactive intestinal peptidinoma, gastrin, glucagon, and vasoactive intestinal peptide secretion, respectively, can be noted [1]. If the pancreatic neuroendocrine tumor is nonfunctioning the clinical presentation is mainly non-specific and is related to the local mass effect [2]. Those patients present with abdominal pain, loss of weight, nausea, symptoms of obstructive jaundice, etc. 

## Case presentation

A 49-year-old female with a background history of poorly controlled type 2 diabetes mellitus for 7 years complicated with peripheral neuropathy, currently on oral hypoglycaemics along with dyslipidemia and bronchial asthma for 19 years on regular inhaler therapy presented with a history of back pain and gastroesophageal reflux symptoms for 3 months duration. 

Back pain was of gradual onset and it was a pain of moderate severity. Diurnal variation of the pain was not noted and no exacerbating or relieving factors for the pain were not noted either. The pain did not seem to radiate to any other area and didn’t seem to progressively worsen over time either. She denied pain affecting her activities of daily living but due to the persistent nature of the pain she has thought of seeking medical advice. During the course of illness, she has taken simple analgesics to which the pain seems to have slightly responded but not completely. 

Along with back pain, she denied a history of diarrhea, loss of weight, loss of appetite, flushing suggestive of carcinoid syndrome, erythematous skin rashes suggestive of glucagonoma, recurrent hypoglycaemic spells suggestive of insulinoma, no unilateral lower limb pain and swelling suggestive of deep vein thrombosis (DVT), no past history or present history of gall bladder calculi, no other joint symptoms or focal neurological deficit, and abdominal pain, no recurrent severe muscle weakness. She has had no similar symptoms before and has no family history of neuroendocrine disorders as well. The onset of her gastroesophageal regurgitant symptoms was around the same time she developed her back pain but she has not sought any medical advice to that. She had a past surgical history of drainage of a frontal cerebral abscess 22 years back, excision of an abdominal wall lump 17 years back, and a history of right superior percutaneous nephrolithotomy done 8 years back. However, she has had no significant surgical complications after any of the above surgeries. No history of known allergies. She had no history of alcohol consumption and no exposure to passive smoke either. 

Upon examination, she was an averagely built lady whose appearance was consistent with her chronological age. She had a BMI of 22 kg/m2. She was afebrile, there was no conjunctival pallor, plethora, or icterus noted. There were no skin rashes or growths noted during the general examination. No generalized lymphadenopathy was noted. She had a pulse rate of 82 beats per minute with a blood pressure of 130/80 mmHg. She had a dual rhythm with no pulmonary stenosis/tricuspid regurgitant murmurs in specific and the respiratory system examination was unremarkable. Upon neurological examination, there was no spinal tenderness, tone, power, reflexes were normal, and sensory, and joint position sensation was intact. There were no tender areas noted during the back examination and the modified Schober’s test was negative. Abdominal examination was unremarkable with no organomegaly, free fluid, or palpable gallbladder.

**Figure 1:**
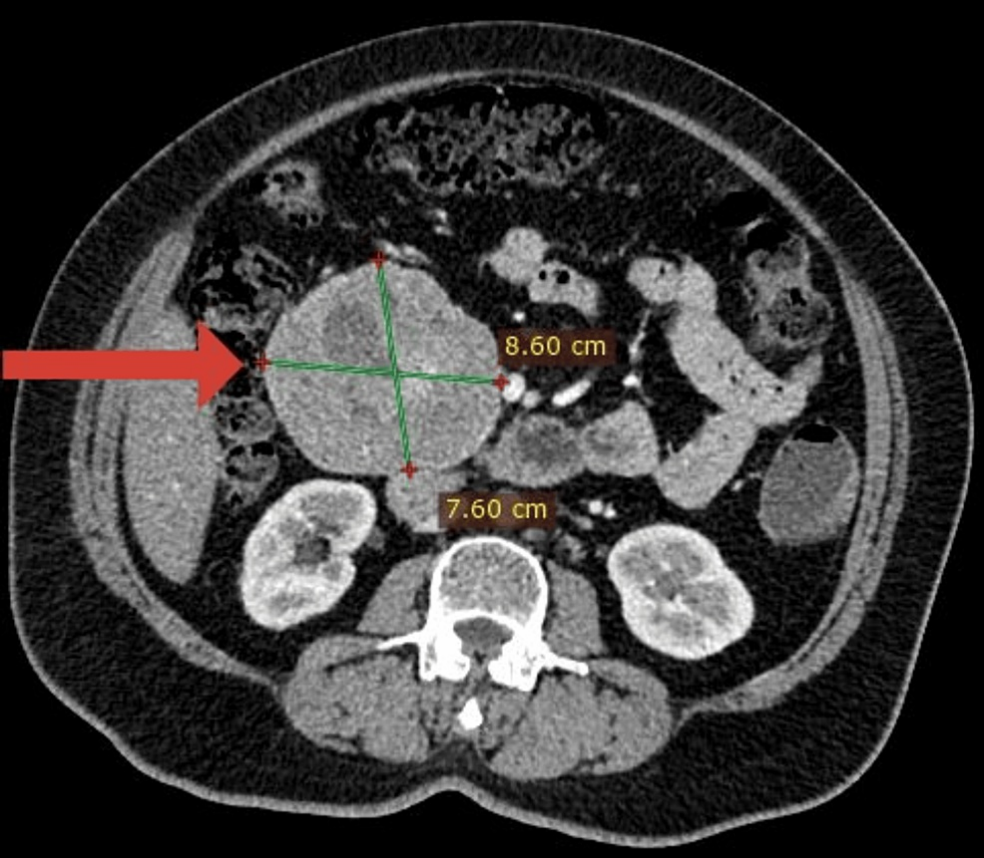
A well-defined mixed solid and cystic dense mass lesion measuring 7.9cm * 8.7cm * 10.2 cm in the retroperitoneum anterior to the 2nd part of the duodenum extending inferiorly and anteriorly to the aorta/inferior vena cava.

In view of arriving at a diagnosis, serial investigations were performed as follows (Table 1). Based on the investigations a diagnosis of pancreatic neuroendocrine tumor was made with no local infiltration or distant metastases (Figure 1). Somatostatin receptor scintigraphy (SRS) could not be done due to limitations in the available facilities. Surgical intervention was planned and pancreaticoduodenectomy (Whipple’s procedure) was done. 

**Table 1:** Investigations

Investigation		Pre-operative Investigation Results	Post-operative Investigation Results	Reference Range
Blood Hematology, Coagulopathy and Biochemistry Tests	White Cell Count (×10^9^/L)	4.37	7.4	4 - 11
	Haemoglobin (g/dL)	12.5	7	12 - 14
	Platelet Count (×10^9^/L)	257	6	150 - 400
	International Normalizing Ratio (INR)	1.27	0.99	<1
	Activated partial thromboplastin time (sec)	-	28.2	21 - 35
	Reticulocyte Count (%)	-	3.12	0.5 – 2.5
	Aspartate transaminase (U/L)	31	27.3	<40
	Alanine transaminase (U/L)	50	43.8	<40
	HBA1C (%) (Haemoglobin A1C)	8.3	-	<7
	Fasting Blood Sugar (mg/dL)	137	141	<126
Serum Electrolytes	Serum Sodium (mmol/L)	139	-	135 - 145
	Serum Potassium (mmol/L)	4.3	-	3.5 - 5.5
Tumor Markers	Cancer Antigen (CA) 19.9 (U/ml)	22.2	-	0 - 37
Immuno histochemistry and staining	Chromogranin A (µg/L)	103.98	255.9	<100
	Synaptophysin	+	+	
	Ki67 protein	<1%	-	
	Beta Catenin	Not available	-	
	Urine *5*-*Hydroxyindoleacetic acid* (5-HIAA)	Negative	-	
Bone Marrow Biopsy and histology	Mildly hypercellular with no abnormal cells. Normal cell maturation sequence. No evidence of hemopoietic or nonhemopoietic cell infiltrates.			
Trephine Biopsy	Peripheral thrombocytopenia with hypercellular megakaryopoiesis is suggestive of immune thrombocytopenia in a background of reactive bone marrow secondary to infective/inflammatory process			
Peripheral Blood Smear (PBS)	Red Blood Cells - Normocytic Normochromic Cells No Fragmented Cells Population of hypochromic microcytic cells White Blood Cells – Neutrophil Leucocytosis Toxic Changes with Left Shift			
Histopathology report of the Tumor	Neuroendocrine tumor of the head of the pancreas in stage pT3			
Imaging Studies	Ultrasound Scan of the Abdomen	There is a retroperitoneal mass measuring 2.5*2.3cm seen in the right abdomen antero inferiorly to the right kidney. Right kidney and pancreas appear normal.		
	Contrast Enhanced Computed Tomography Scan of the Abdomen (CECT Abdomen)	A well-defined mixed solid and cystic dense mass lesion measuring 7.9cm *8.7cm *10.2 cm in the retroperitoneum anterior to the 2^nd^ part of the duodenum extending inferiorly and anteriorly to the aorta/inferior vena cava. Arise from the medial surface of the head of the pancreas and uncinate process of the pancreas		
	2 Dimensional Echocardiography	Diabetic Heart Disease with grade 1 diastolic dysfunction Ejection Fraction – 70% Cardiac Chambers are normal		

From postoperative day 1 she was noted to have severe thrombocytopenia which was progressively worsening over time along with a reduction in hemoglobin levels. Postoperative bleeding was suspected at initial stages and the investigations done postoperatively suggested the possibility of disseminated intravascular coagulation (DIC) being the causative factor for the above presentation. Among the other differential diagnoses that were postulated at this stage included drug-induced thrombocytopenia or reduction of platelets secondary to sepsis.

Since her hemoglobin was low, she was given two units of red cell concentrates along with two units of platelets. She did not show any superficial bleeding manifestations either. Furthermore, the bone marrow biopsy done showed hypercellular megakaryopoiesis, hence excluding the possible differential diagnoses, a diagnosis of immune thrombocytopenia was made and she was initiated on oral prednisolone 40 mg daily dose. It was tapered over a 4-week duration as there was a rise in the platelet count following prednisolone treatment. 

During the subsequent clinic visits, 6 months following surgery, she was found to have ecchymotic patches mainly distributed over the chest. During that presentation, she was found to have a very low platelet count with the minimum count being 3*10^9^/cumm^3^. During this presentation repeated ultrasound scan of the abdomen was done and it showed no residual mass in the pancreatic region but a hepatomegaly of 16.9 cm along with grade 2 fatty liver was noted. Neither any other definitive intra-abdominal mass nor para-aortic lymphadenopathy was noted.

Hence the diagnosis of immune thrombocytopenia was well established and she was initiated on intravenous methylprednisolone 1 g daily three doses along with an increased dose of oral prednisolone 60 mg to be taken daily. A few days later, a rise in platelet count was noted. She was also given eltrombopag, which is a thrombopoietin receptor agonist, 50 mg daily to stimulate thrombocyte regeneration.

## Discussion

Among all pancreatic tumors, pancreatic neuroendocrine tumors account for a very small proportion [2]. They are epithelial neoplasms that have a specific neuroendocrine differentiation. The majority of these tumors are well differentiated and a slow progression over time is noted [3]. The functionality or non-functionality of the said pancreatic neuroendocrine tumors is determined by the presence of specific clinical manifestations [1]. Considering the presentation of this index case, she presented with only back pain, which is a non-specific symptom that can be due to any intra-abdominal mass, and she did not have any hormonal symptoms; hence, it was found to be non-functional in origin. Epidemiologically, no significant differences are noted in the incidence of pancreatic neuroendocrine tumors among different geographical locations, and no differences in gender or race are also noted [2]. These tumors characteristically present in the sixth to seventh decades of life, although the patient described in this report presented earlier [2,4]. 

Among the risk factors for the development of pancreatic neuroendocrine tumors, diabetes mellitus, smoking, genetic susceptibility, and a previous history of chronic pancreatitis play a major role [1]. In this case, poorly controlled diabetes mellitus was a significant risk factor for the development of this disease. 

Since this is a neuroendocrine tumor with a foregut origin, the European Neuroendocrine Society suggests the staging and grading of these tumors be done according to the proliferative potential of the tumor, which can be measured based on the Ki-67 index [4,5]. In the usual setting, neuroendocrine tumors are smaller in size (<2cm) at the time of presentation, hence the compressive symptoms that can occur due to mass effect on the adjacent organs are sparse. But, in this particular case, she had a significant tumor size, and it may be the reason for the back pain with which she presented. 1-2% of pancreatic neuroendocrine tumors are related to familial disorders including MEN syndrome, tuberous sclerosis and type 1 neurofibromatosis [3]. In the clinical history of this patient, such diagnoses in the family were not noted.

Diagnosis of pancreatic neuroendocrine tumors is based on imaging modalities and tumor markers [6,7]. Imaging is not very useful in a setting where the tumor size is less than 5 mm [6]. However, in this patient, since the tumor was advanced at the time of presentation, the existing imaging modalities, including an ultrasound scan of the abdomen and a CT scan of the abdomen, were useful in determining the exact tumor size and location. Endoscopic ultrasound scanning is beneficial for the detection of small tumors, and it has the added benefit of obtaining biopsies when necessary to determine the histological type of the tumor [4,8]. Yet this was not performed in this patient as it was opted for surgical management in the first place due to the increased tumor size.

Tumor markers are beneficial in the diagnosis, determining the prognosis, and therapeutic options. Chromogranin A (CgA) is a non-specific tumor marker to detect neuroendocrine tumors. It is usually elevated in 50-100% of individuals with neuroendocrine tumors [7]. The specificity and sensitivity of this marker in detecting neuroendocrine tumors are 73% and 95%, respectively. CgA is more sensitive for foregut neuroendocrine tumors while 5-Hydroxyindoleacetic acid (5-HIAA) is more specific for hindgut neuroendocrine tumors. The depth of tumor burden and disease progression can be determined by this tumor marker. Yet the importance of this tumor marker in non-functional tumors is controversial [7]. 

Considering the management options available, surgical resection is the most well-known curative option available for these patients [8]. Octreotide can be used as a management option in patients who are not fit for surgical resection. The place for curative surgery is advisable for patients with nonfunctional neuroendocrine tumors given that the local resectability is possible and no distant metastasis is apparent. This was seen in this index case as well, hence Whipple’s procedure was carried out in this patient. Response to chemotherapy is not well proven for pancreatic neuroendocrine tumors. Among the complications reported following the excision of the pancreatic neuroendocrine tumor, thrombocytopenia is a recognized complication, mainly after chemotherapeutic drug administration and following transarterial chemoembolization. 

Thrombocytopenia can occur as a result of myelosuppression, due to sepsis, as a result of an adverse reaction to pharmacological management, due to disseminated intravascular coagulation, etc. [9,10]. In this case, the patient did not have thrombocytopenia prior to the surgical intervention, and no chemotherapeutic drug was advocated as a part of management. Hence, the possibility of myelosuppression secondary to chemotherapy is not applicable to this case. The imaging studies done prior to the surgery did not show evidence of local or distant metastases. Hence myelosuppression due to tumor invasion is not a possibility in this case. Myelosuppression due to other reasons too was not considered as there was increased platelet turnover noted in the bone marrow biopsy. Increased peripheral utilization or destruction of platelets as a consequence of disseminated intravascular coagulation (DIC) is a possibility in this case. She had reduced hemoglobin levels post-surgery but no evidence for secondary hemorrhage was noted. No intrabdominal bleeding was noted in imaging studies, no fragmented red cells were in the blood picture, the ROTEM (rotational thromboelastometry) study was normal, and the fibrin degradation study was negative. Hence, DIC was unlikely.

Secondary autoimmune thrombocytopenia is when immune-mediated thrombocytopenia resembles idiopathic thrombocytopenia due to different causes. Immune-mediated platelet destruction can be a causative factor for thrombocytopenic syndromes noted in patients with solid tumors. Yet this can only be diagnosed by antibody or kinetic verification tests, which were not performed in this case. Autoimmune thrombocytopenia is found in patients with solid tumors, including lung, prostate, breast, kidney, ovaries, and testis [10]. They will show elevated levels of platelet-associated IgG levels. Similar elevations of platelet-associated IgG levels are noted in patients with drug-induced thrombocytopenia as well. In this index case, she was given third-generation cephalosporins as a prophylactic measure to prevent surgical site infection. Hence, even though there is no evidence for that, still there is a possibility that she developed drug-induced thrombocytopenia secondary to this drug. On the contrary, she developed a relapse 6 months after the surgery with full-blown skin manifestations of thrombocytopenia without administration of new medication; hence, drug-induced thrombocytopenia was excluded. 

Considering the fact that she responded to corticosteroids, the diagnosis of immune thrombocytopenia was made in this patient. Following treatment with prednisolone, a rise in platelet count was noted. It was further accentuated by the fact that the second refractory episode, which was resistant to steroids, was well controlled with eltrombopag. Among the other available options for ITP are plasmapheresis and IVIG. Second-line options include eltrombopag (which was administered during the second episode), rituximab, and splenectomy. Third-line options are mostly immunosuppressants such as azathioprine or cyclosporine [9].

## Conclusions

Non-functional pancreatic neuroendocrine tumors are a rare type of malignancy. Surgical resection is the mainstay of management offered to these patients. Among the complications noted in these patients, thrombocytopenia is a manifestation that could be a consequence of myelosuppression, drug-induced, disseminated intravascular coagulation, or as a part of immune-mediated mechanisms. In this case, the patient developed immune thrombocytopenia following surgical enucleation of a neuroendocrine tumor that responded to steroids. Active surveillance and prompt intervention are mandatory to prevent these complications from becoming life-threatening in due course. 
